# Olfaction in patients with Parkinson’s disease: a new threshold test analysis through turning points trajectories

**DOI:** 10.1007/s00702-021-02387-z

**Published:** 2021-07-30

**Authors:** Maria Paola Cecchini, Elisa Mantovani, Angela Federico, Alice Zanini, Sarah Ottaviani, Carla Masala, Michele Tinazzi, Stefano Tamburin

**Affiliations:** 1 Section of Anatomy and Histology, Department of Neurosciences, Biomedicine and Movement Sciences, University of Verona, Strada le Grazie, 8, 37134 Verona, Italy; 2 Section of Neurology, Department of Neurosciences, Biomedicine and Movement Sciences, University of Verona, Piazzale Scuro 10, 37134 Verona, Italy; 3 Section of Neurology, Verona University Hospital, Verona, Italy; 4 Department of Biomedical Sciences, Section of Physiology, University of Cagliari, Cagliari, Italy

**Keywords:** Chemosensory function, Cognition, Olfaction, Threshold test, Parkinson’s disease (PD)

## Abstract

Olfactory deficit is a widely documented non-motor symptom in Parkinson’s disease (PD). Abnormal turning points trajectories through olfactory threshold testing have been recently reported in patients with olfactory dysfunction, who seem to adapt faster to olfactory stimuli, but data on PD patients are lacking. The aim of this study is to perform olfactory threshold test and explore the turning points trajectories in PD patients in comparison to normal controls. We recruited 59 PD patients without dementia, and no conditions that could influence evaluation of olfaction and cognition. Sixty healthy subjects served as controls. Patients and controls underwent a comprehensive olfactory evaluation with the Sniffin’ Sticks extended test assessing threshold, discrimination and identification and a full neuropsychological evaluation. Besides, threshold test data were analyzed examining all the turning points trajectories. PD patients showed a different olfactory threshold test pattern, i.e., faster olfactory adaptation, than controls with no effect of age. Normosmic PD patients showed different olfactory threshold test pattern, i.e., better threshold score, than normosmic controls. Visuospatial dysfunction was the only factor that significantly influenced this pattern. Olfactory threshold trajectories suggested a possible adaptation phenomenon in PD patients. Our data offered some new insights on normosmic PD patients, which appear to be a subset with a specific psychophysical profile. The analysis of the turning points trajectories, through an olfactory threshold test, could offer additional information on olfactory function in PD patients. Future larger studies should confirm these preliminary findings.

**Supplementary Information:**

The online version contains supplementary material available at 10.1007/s00702-021-02387-z.

## Introduction

Olfactory dysfunction is a highly prevalent non-motor feature of Parkinson’s disease (PD) that may occur several years before the onset of motor symptoms, with a prevalence of 45–98% during all disease stages (Haehner et al. 2009,2011; Doty 2012; Rahayel et al. 2012; Fullard et al. 2017; Marin et al. 2018) and an idiopathic smell deficit is a possible marker of future PD (Heinzel et al. 2019; Haehner et al. 2019). Olfactory deficits in PD involve several components of odor perception, i.e., identification, discrimination, and detection threshold (Nielsen et al. 2018). While olfactory identification and discrimination domains were found to better differentiate PD (Rahayel et al. 2012; Zhao et al. 2020), and other neurodegenerative disorders (Whitcroft et al. 2016) vs. controls, data on olfactory threshold appear to be less consistent, being reported as both relatively spared (Whitcroft et al. 2016) and affected in PD (Quinn et al. 1987; Bovi et al. 2010; Rahayel et al. 2012; Park et al. 2018). The ratings of suprathreshold evaluation of perceived odor intensity were decreased in PD vs. controls with no apparent link to the dopaminergic system activity (Doty et al. 2014). A specific study in elderly PD patients showed that detection threshold scores to three different stimuli could discriminate elderly patients and controls, being also significantly different between PD patients with good vs. impaired autonomy (Foguem et al. 2018). These data underscore the need to further and more deeply investigate odor threshold in PD. Indeed, odor threshold measurement is important in assessing olfactory adaptation, a sensory process operating both at peripheral and central levels (Pellegrino et al. 2017; Lawson et al. 2018). Olfactory adaptation is defined as reduced perceived intensity of an odor after repeated or prolonged odorant exposure (Dalton 2000). After a prolonged odorant exposure, odor threshold measurement indicates the adaptation to that odorant, so that an increased detection threshold and a transitory inability to perceive intensity of an odor occur; then, olfactory sensitivity progressively recovers (Dalton 2000; Stuck et al. 2014; Pellegrino et al. 2017). This physiological mechanism allows adaptation to environmental changes (Störtkuhl et al. 1999). Olfactory adaptation is considered critical for survival, making the subject ready to changes in environmental olfactory stimuli, and early identification of abnormalities in this process is highly recommended (Brai and Alberi 2018).

Few studies explored olfactory adaptation in clinical settings (Pellegrino et al. 2017), e.g., in autism spectrum disorders (Tavassoli and Baron-Cohen 2012; Kumazaki et al. 2019) or multiple chemical sensitivity (Andersson et al. 2009, 2016), with contrasting findings.

Recently, a study of the trajectory turning points in odor threshold test, as an approximation of olfactory adaptation, was explored in a large database of patients with olfactory deficits of different severity (i.e., hyposmia, functional anosmia) to different causes, except neurodegenerative disorders (Chen et al. 2020). The threshold test explores the concentration at which a target odor is reliably detected among triplets of pens, of which two contain an odorless solution and one the odorant. This test avoids olfactory adaptation through an adequate interstimulus interval (e.g., 30 s) (Doty et al. 1986), so that this procedure is not the gold standard for measuring adaptation. Nevertheless, Chen et al. (2020) showed that weak repeated stimuli could induce olfactory adaptation in patients with olfactory dysfunction, in that they adapt faster to olfactory stimuli than healthy controls during the threshold test administration. Thus, they concluded that olfactory threshold trajectories analysis may be a useful indicator of olfactory adaptation in clinical practice (Chen et al. 2020).

To the best of our knowledge, no study has explored olfactory adaptation by means of the threshold test trajectories analysis in PD patients, so far. To add more information on this topic, we performed a detailed threshold test analysis in idiopathic PD patients. For this purpose, we recruited a group of PD patients and age- and sex-matched healthy controls, who underwent a comprehensive olfactory evaluation by means of the Sniffin’ Sticks extended test (SSET), a validated smell test (Hummel et al. 2007; Oleszkiewicz et al. 2019). We also analyzed the SSET threshold data in terms of turning point trajectories (Chen et al. 2020). Data were further compared to those of an older PD cohort. Since we previously demonstrated that mild cognitive impairment (MCI) could negatively influence olfactory identification performance (Cecchini et al. 2019), patients underwent a thorough cognitive evaluation and were stratified according to the presence of MCI and the involvement of single cognitive domains. The effect of demographic, clinical, cognitive, and neuropsychiatric covariates on olfactory threshold test was also explored through a multivariate model.

## Methods

### Subjects

We evaluated 135 consecutive PD patients at the Department of Neuroscience, Verona University Hospital, Italy. Inclusion criteria were: (a) diagnosis of idiopathic PD; (b) no PD-associated dementia (Jellinger 2018); (c) no coexisting reasons (e.g., delirium, cerebrovascular disease, head trauma, metabolic abnormalities, medication adverse effects) that could have influenced olfaction and/or cognition (Litvan et al. 2012; Drareni et al. 2020); (d) no other PD-related conditions (e.g., severe motor impairment, psychosis, severe motor fluctuations or dyskinesia, excessive daytime sleepiness) that could have influenced assessment of cognition (Litvan et al. 2012; Federico et al. 2017) and olfaction; (e) no history of ear nose and throat disorders, middle ear surgery, head or face trauma, Bell’s palsy, systemic diseases or any other clinical condition that could have interfered with olfaction and taste evaluation, and (f) no current smoking (Ajmani et al. 2017).

After screening for inclusion criteria (Supplementary Fig. 1), 59 patients (25 women, 34 men; age: 66.5 ± 10.9 years, median 69, interquartile range, IQR 57–74.5) were included in the study. PD patients were divided into two groups, namely middle age PD (MA-PD; age < 70; *N* = 31, 11 women, 20 men; age: 58.2 ± 8.5 years, median 57, IQR 54–66.5) and older age PD (OA-PD; age ≥ 70, *N* = 28, 14 women, 14 men; age: 75.7 ± 3.7 years, median 76, IQR 73–78.5). First, middle age PD group was compared to age- and sex-matched healthy controls to explore differences in olfactory threshold test trajectories related to PD. Then, a further analysis included both PD groups to explore consistency of the findings in older patients.

PD motor symptoms were measured with the modified Hoehn–Yahr (H–Y) scale and the Movement Disorder Society unified Parkinson’s disease rating motor subscale (UPDRS-III). Total levodopa equivalent daily dose (LEDD, mg) was calculated according to conversion formulae (Tomlinson et al. 2010).

Sixty healthy controls (33 women, 27 men; *p* = 0.17 vs. patients; age 56.9 ± 9.6 years, median 55, IQR 49–63; *p* < 0.001 vs. PD patients; *p* = 0.43 vs MA-PD) were screened for cognition with the Montreal cognitive assessment (MoCA) and underwent a detailed clinical history collection to rule out conditions that could have interfered with olfaction and taste evaluation (points e, f) of inclusion criteria for patients. All control subjects were extracted from an archived database of volunteers evaluated at the Department of Neurosciences, Biomedicine and Movement Sciences, University of Verona and the Department of Biomedical Sciences, University of Cagliari through public announcements.

The study was approved by Verona University Hospital ethical committee. Participants gave written consent prior to inclusion in the study, which was conducted according to the 1964 Declaration of Helsinki and its later amendments.

### Olfactory evaluation

Olfaction was assessed in a well-ventilated room with the SSET (Burghart, Wedel, Germany), a validated test that consists of odor-containing felt-tip pens and based on a forced-choice paradigm (Hummel et al. 2007; Oleszkiewicz et al. 2019). The SSET is composed of three subtests, namely odor threshold (i.e., detecting the lowest concentration), odor discrimination (i.e., separating a specific odor from others) and odor identification (i.e., recognizing and naming a specific odor). For the threshold and discrimination test, subjects are blindfolded to prevent the visual identification of target pens. The sum of the SSET odor threshold, discrimination and identification scores (TDI score) defines the olfactory performance of subject as normosmia (TDI Score ≥ 30.75), hyposmia (TDI < 30.75 and > 16), and functional anosmia (i.e., total loss or minimal residual smell perception; TDI ≤ 16) (Kobal et al. 2000; Hummel et al. 2007; Weintraub et al. 2015).

### Threshold test procedure

The threshold test explores the concentration at which a target odor (n-butanol) is reliably detected among triplets of pens, of which two contain an odorless solution and one the odor. Subjects are asked to identify the odor-containing pen each time. Triplets of pens are randomly presented, and the answers are recorded by means of a forced-choice procedure. The test consists of sixteen dilutions, prepared in a geometric series starting from a 4% n-butanol solution (dilution ratio 1:2 in deionized water as solvent).

Starting with the lowest n-butanol concentration, a staircase paradigm is used. Reversal of the staircase (i.e., the presentation of the triplet with the next lower odor concentration) is started when the odor-containing pen is correctly identified in two successive trials (starting point). Then, when subjects give an incorrect answer, the triplet with the next higher odor concentration is presented and thus, the staircase is reversed again (defining different turning points). Testing is completed after seven reversals of the staircase. Odor threshold final score is obtained with the mean of the last four out of seven turning points of the staircase. The threshold score could range from 1 to 16, the higher the score, the better the olfactory detection performance.

### Threshold test trajectories analysis

All the turning points trend trajectories were analyzed according to the previously reported procedure (Chen et al. 2020). The difference between the first turning point (starting point) and the final score of detection threshold, and the number of trials taken to reach the final threshold score were also calculated (Chen et al. 2020).

### Cognitive assessment

All patients were in a stable ON condition and underwent the mini mental state examination (MMSE), MoCA and a comprehensive 15-test neuropsychological battery that were performed by an expert neuropsychologist (AF) in a quiet room (Goldman et al. 2015; Federico et al. 2015, 2017). The diagnosis of MCI-PD was based on the Movement Disorder Society level II criteria, which stipulate a cognitive battery including at least two tests for each of the five cognitive domains (i.e., memory, attention, executive function, visuospatial function and language) and the abnormality of at least two tests (Litvan et al. 2012). Memory was examined with the Rey’s auditory verbal learning immediate and recall tests (Carlesimo et al. 1996). Attention and working memory were assessed with the digit span forward (Mondini et al. 2011), attentional matrices parts I and II (Della Sala et al. 1992), and trail making test part A (Mondini et al. 2011). Executive function was explored with the frontal assessment battery (Appollonio et al. 2005), phonemic fluency test (Mondini et al. 2011) and the Stroop task (Brugnolo et al. 2016). Visuospatial function was assessed with the Benton judgement of line orientation test (Benton et al. 1978), the intersecting pentagons derived from the MMSE (Federico et al. 2017) and the clock copying test (Goldman et al. 2015). Language was evaluated with the short form of the Boston naming test (Fastenau et al. 1998), object naming test and verb naming test (Capasso and Miceli 2001). MCI was defined as single- or multi-domain, according to the number of cognitive domains involved (Litvan et al. 2012).

### Neuropsychiatric assessment

Depression was assessed with the Hamilton depression rating (HAD) scale. Apathy was evaluated with the apathy evaluation self-report (AES-S) scale (Marin et al. 1991).

### Statistical analysis

The normality of distribution was analyzed with the skewness–kurtosis test. Continuous variables were explored with *t* test and non-parametrical Mann–Whitney *U* test according to the distribution normality. Pearson’s χ^2^ test with Yates’ correction was applied to dichotomous variables. Two-way repeated measures (RM) ANOVA with within-group factor Turning Point (seven levels) and between-group factor Group (two levels: MA-PD patients, controls) and post hoc *t* test with Bonferroni’s correction were used to compare the odor threshold trajectory turning points in patients and controls. One-way ANOVA and post hoc *t* test with Bonferroni’s correction were applied to compare TDI score between groups (three levels: MA-PD, OA-PD, controls). Multi-way RM-ANOVA with within-group factor Turning Point (seven levels), between-group factor Group (two levels: PD patients, controls), Gender (two levels: women, men), and Age (continuous variable) as covariates and post hoc *t* test with Bonferroni’s correction was applied to compare patients and controls. Multi-way RM-ANOVA with within-group factor Turning Point (seven levels), between-group factors MCI and cognitive domains (two levels: yes/no) and Olfactory Status (three levels: normosmia, hyposmia, functional anosmia), H–Y, UPDRS-III, LEDD, HAD and AES-S as covariates and post hoc *t* test with Bonferroni’s correction was applied to compare the different threshold patterns in PD patients according to the motor, pharmacological, cognitive, neuropsychiatric, and olfactory *status*. *p* < 0.05 (two tailed; with Bonferroni’s correction for multiple comparisons) was taken as the significance threshold for all the tests.

## Results

### PD clinical features

Clinical features did not differ between MA-PD and OA-PD groups (Table 1).

**Table 1:** Clinical characteristics of PD patients

Variable	MA-PD (*N* = 31)	OA-PD (*N* = 28)	*p*
PD duration (years)	4.5 ± 3.9; 3–5	5.6 ± 4.7; 3–6	0.33
H–Y (1–5)	1.7 ± 0.8; 1–2	1.7 ± 0.8; 1–2	0.35
MDS UPDRS-III (0–132)	18.4 ± 10.7; 11.5–28.5	20.5 ± 13.8; 12–28	0.58
Treatment			
LD (yes/no)	22/9	25/3	0.16
DA (yes/no)	15/16	8/20	0.12
MAO-I (yes/no)	13/18	10/18	0.62
Total LEDD (mg)	645 ± 530; 310–780	458 ± 191; 325–745	0.34
MMSE (0–30)	27.3 ± 3.6; 26–28.5	26.4 ± 3.8; 25–28	0.35
MCI (multidomain/single domain/no)	16/7/8	11/9/8	0.60
Involved cognitive domain^a^			
Memory (yes/no)	4/27	4/24	0.88
Attention and WM (yes/no)	6/25	11/17	0.09
Executive function (yes/no)	13/18	18/10	0.09
Visuospatial function (yes/no)	6/25	3/25	0.36
Language (yes/no)	0/31	0/27	–
HAD (0–52)	7.2 ± 6.2; 3–10	6.2 ± 4.6; 3–10	0.48
AES-S (18–72)	29.9 ± 10.9; 18.5–39	29.7 ± 11.8; 19–39	0.67

Continuous data are presented as mean ± SD, interquartile range

*AES-S* apathy evaluation self-report scale, *DA* dopamine agonist, *HAD* Hamilton depression rating scale, *H–Y* modified Hoehn and Yahr staging scale, *LD* levodopa, *LEDD* levodopa equivalent daily dose, *MA-PD* middle age PD (age < 70), *MAO-I* monoamine oxidase inhibitor, *MCI* mild cognitive impairment, *MDS UPDRS-III* Movement Disorders Society unified Parkinson's disease rating scale part III, *MMSE* mini mental state examination, *OA-PD* older age PD (age ≥ 70), *PD* Parkinson’s disease, *PD-MCI +* PD patients with MCI, *PD-MCI−* PD patients without MCI, *WM* working memory, *MA-PD* middle age PD (age < 70)

^a^The cognitive domain was considered as involved when at least one neuropsychological test of that domain was abnormal

### Olfactory evaluation

TDI score was significantly worse in PD patients (mean 20.7 ± 7.1, median 19.5, IQR 12–31.4) than controls (mean 32.3 ± 1.7, median 32, IQR 31.1–33.5; *p* < 0.001), being all the subjects in the latter group classified as normosmic ones. ANOVA indicated that TDI Score was significantly different between groups (*F*_[2,118]_ = 64.3,* p* < 0.001), in that MA-PD (22.1 ± 7.6) and OA-PD patients (19.2 ± 6.3) exhibited significantly lower values (i.e., worse olfaction) than controls (post hoc: *p* < 0.001 for both comparisons). No significant SSET differences were found comparing MA-PD vs. OA-PD groups (*p* = 0.12). According to TDI score, 8 PD patients had normosmia (age: 60.5 ± 13.9 years), 31 showed hyposmia (age: 66.7 ± 11.0 years), and 20 had functional anosmia (age: 68.9 ± 11.0 years; *p* = 0.19).

Detection threshold final score was significantly worse in PD patients (5.0 ± 3.4, median 4.5, IQR 1.8–7.3) than controls (6.1 ± 2.4, median 5.8, IQR 4.5–7.8; *p* = 0.022), but not significantly different between MA-PD (5.0 ± 3.2, median 4.8, IQR 13.–11.0) and OA-PD (MA-PD: 4.9 ± 3.7, median 4.0, IQR 1.8–10.4;* p* = 0.71).

### Threshold test trajectories analysis in PD vs. controls

**Fig. 1:**
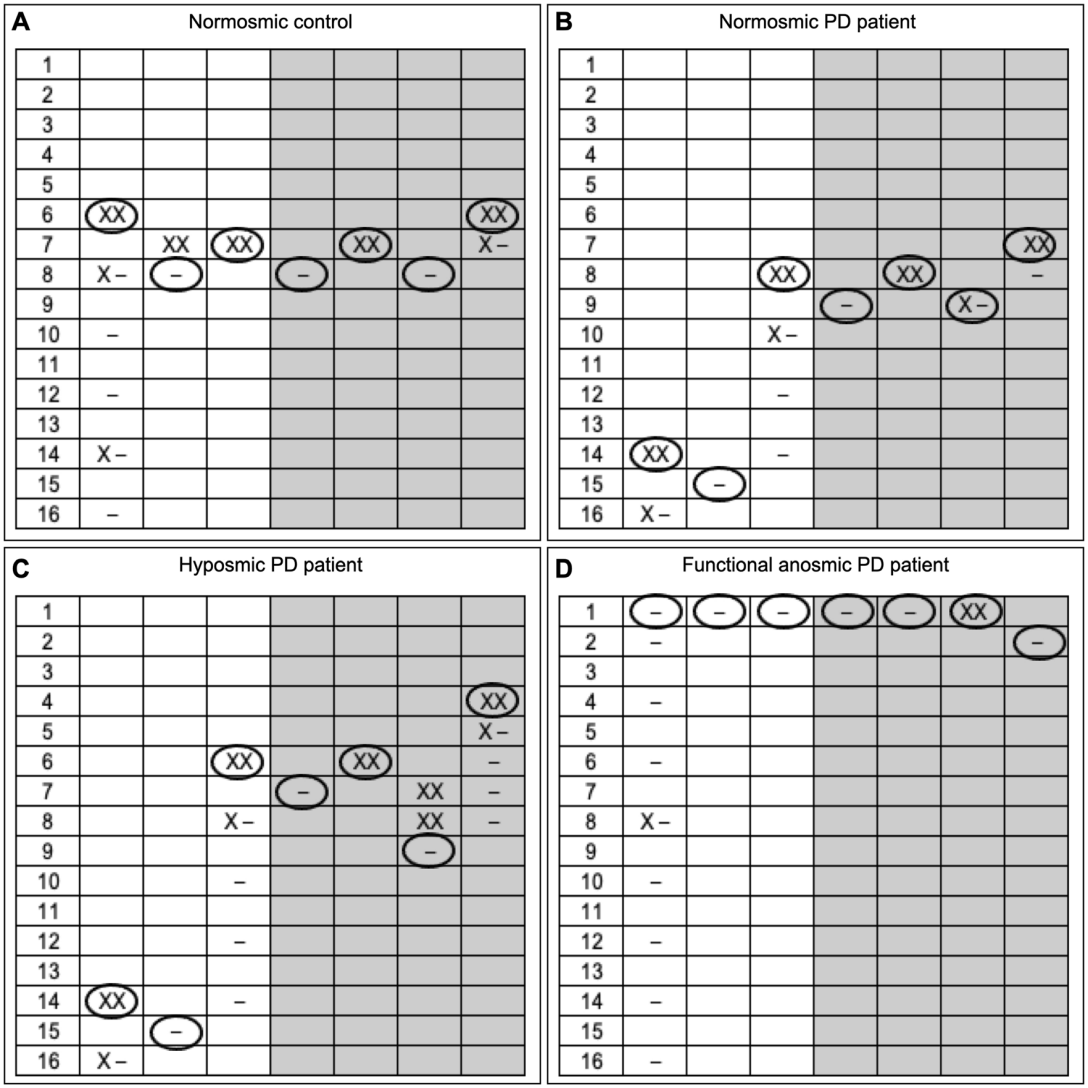
Typical examples of trajectories of olfactory threshold test in a normosmic control (panel **A**) and three patients with Parkinson’s disease (PD), i.e., a normosmic PD patient (panel **B**) a hyposmic PD patients (panel **C**) and a PD patient with functional anosmia (panel **D**). The symbol ‘X’ marks correct responses, while the symbol – marks incorrect ones. Ellipses around the boxes mark the turning points. Starting with the lowest n-butanol concentration (pen number 16), a staircase paradigm is used. Reversal of the staircase (i.e., the presentation of the triplet with the next lower odor concentration) is started when the odor-containing pen is correctly identified in two successive trials (starting point). Then, when subjects give an incorrect answer, the triplet with the next higher odor concentration is presented and thus, the staircase is reversed again to explore different turning points. Testing is complete after seven reversals of the staircase. Odor threshold final score is calculated as the mean of the last four out of seven turning points of the staircase (marked with light gray shade). Higher and lower olfactory threshold score value represents better and worse performance, respectively

The olfactory threshold trajectories were analyzed exploring the seven threshold test turning points. Typical examples are reported in Fig. 1.

**Fig. 2:**
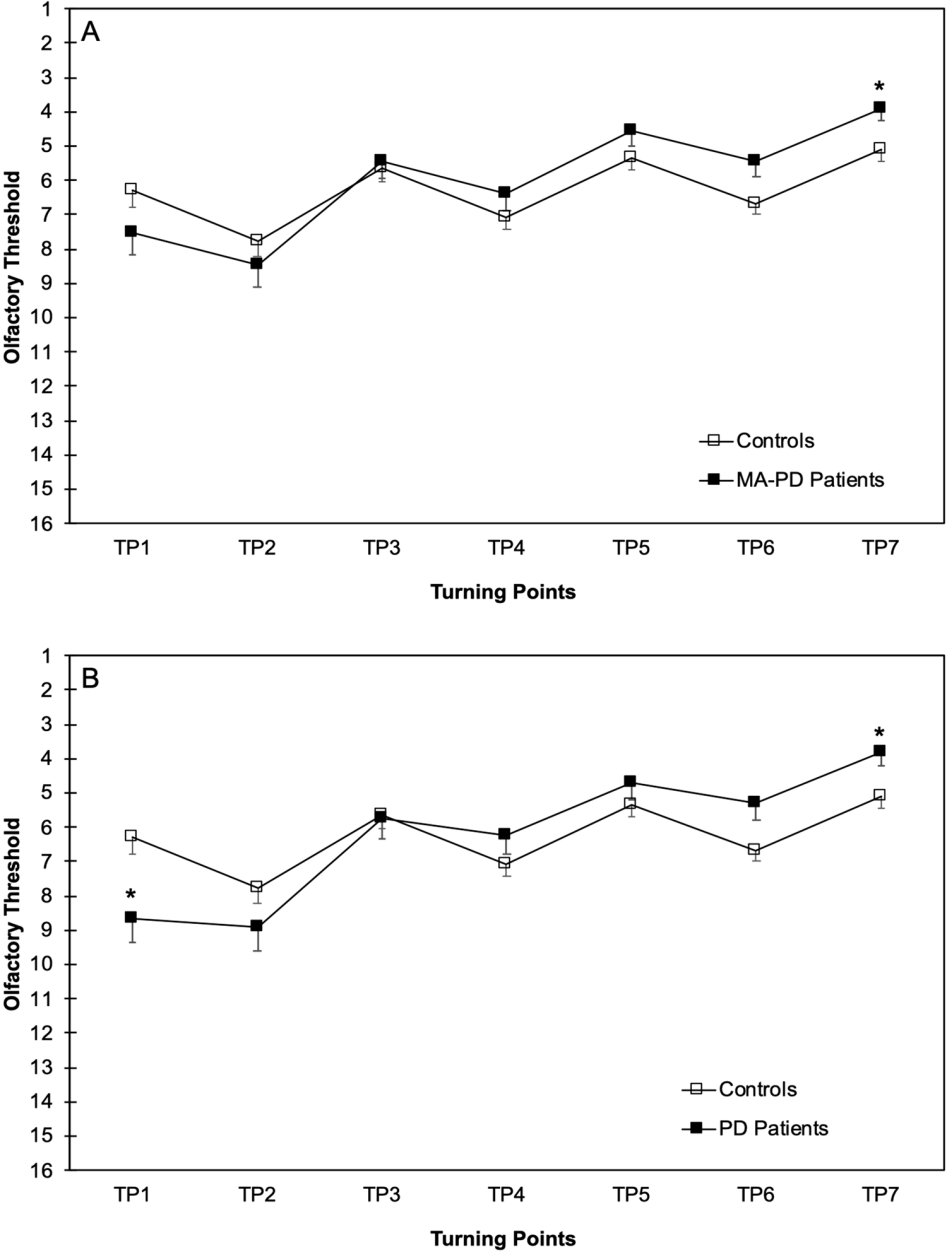
Olfactory threshold at the seven turning points (TP) in controls (*N* = 60; open boxes) and Parkinson’s disease (PD) patients with age < 70 years (MA-PD; *N* = 31; closed boxes; panel **A**) and PD patients (PD; *N* = 59; closed boxes; panel **B**). Higher and lower olfactory threshold score value represents better and worse performance, respectively. *Marks significant patients vs. controls comparison

During the first trials, the threshold turning points in PD patients were better than controls (i.e., higher value), then they were comparable to that of controls since the third turning point, then finally worsened (i.e., PD patients showed lower values than controls) for the last trials (Fig. 2). This pattern indicates faster olfactory threshold adaptation in PD than controls. Two-way RM-ANOVA showed significant effect of Turning Point (*F*_[6,522]_ = 45.1; *p* < 0.001) and significant Turning Point × Group interaction (*F*_[6,522]_ = 6.5; *p* < 0.001), but no effect of Group (*F*_[1,87]_ = 0.2; *p* = 0.62) when comparing MA-PD and controls for odor threshold turning point trajectory. Post hoc analyses showed that detection threshold value was significantly lower in MA-PD patients than controls at the last turning point (*p* = 0.005; Fig. 2A).

A further two-way RM-ANOVA, including MA-PD, OA-PD and controls, showed significant effect of Turning Point (*F*_[6,702]_ = 26.8; *p* = 0.01) and significant Turning Point × Group interaction (*F*_[6,702]_ = 14.7; *p* < 0.001), but no effect of Group (*F*_[1,117]_ = 0.1; *p* = 0.83). Multi-way RM-ANOVA with Age and Gender as covariates confirmed significant effect of Turning Point (*F*_[6,702]_ = 56.8; *p* < 0.001) and significant Turning Point × Group interaction (*F*_[6,702]_ = 4.8; *p* < 0.001), with no effect of Group (*F*_[1,117]_ = 1.0; *p* = 0.33) and showed significant Turning Point × Age interaction (*F*_[6,702]_ = 5.6; *p* < 0.001) but no effect of Age (*F*_[1,117]_ = 2.6; *p* = 0.11). Post hoc analyses showed that detection threshold value was significantly higher in PD patients than controls at the first turning point (*p* = 0.003), while it was significantly lower in patients than controls at the last turning point (*p* = 0.002; Fig. 2B).

The difference between the first turning point and the final threshold score significantly differed when comparing MA-PD patients (2.4 ± 3.6) to controls (0.3 ± 2.6; *p* = 0.002) and PD patients (3.6 ± 4.4) to controls (*p* < 0.001). The number of trials did not significantly differ when comparing MA-PD patients (15.8 ± 2.6) to controls (15.4 ± 2.6; *p* = 0.53) and PD patients (15.9 ± 2.4) to controls (*p* = 0.28).

### Threshold test trajectories analysis in PD according to olfactory status

**Fig. 3:**
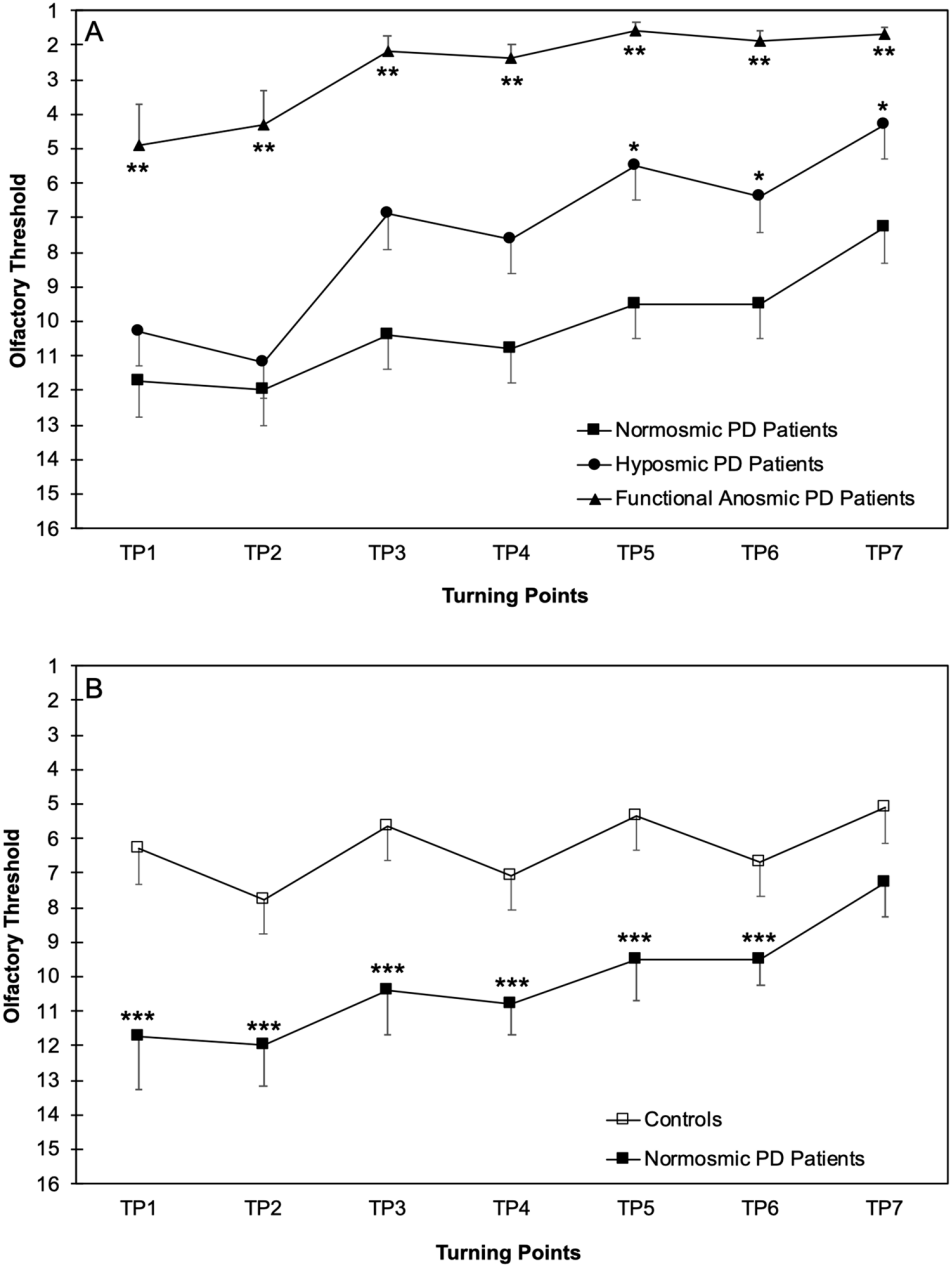
Olfactory threshold at the seven turning points (TP) in Parkinson’s disease (PD) patients according to olfactory function (normosmic: *N* = 8, closed squares; hyposmic: *N* = 31, closed circles; functional anosmic: *N* = 20, closed triangles; panel **A**) and in normosmic PD patients and controls (*N* = 60, open boxes; panel **B**). Higher and lower olfactory threshold score value represents better and worse performance, respectively. *Marks significant hyposmic vs. normosmic PD comparison; **Marks significant functional anosmic vs. other PD subgroups comparison (panel **A**). ***Marks significant normosmic PD patients vs. controls comparison

The turning points trajectories were significantly different in PD patients with different olfactory conditions (i.e., normosmia, hyposmia, functional anosmia), in that olfactory thresholds were worse at all turning points in functional anosmic vs. hyposmic and normosmic PD patients (Fig. 3A). Two-way RM-ANOVA showed significant effect of Olfactory Status (*F*_[2,56]_ = 34.0; *p* < 0.001), Turning Point (*F*_[6,336]_ = 24.5; *p* < 0.001) and significant Turning Point × Olfactory Status interaction (*F*_[12,336]_ = 2.5; *p* = 0.004). Post hoc analyses showed that the detection threshold was significantly lower in functional anosmic PD patients in comparison to the two other PD subgroups at all turning points (*p* < 0.001; Fig. 3A) and in hyposmic vs. normosmic patients at the last three turning points (*p* ranging from 0.001 to 0.002; Fig. 3A). The difference between the first turning point and the final detection threshold score (i.e., the mean of the last four turning points) did not significantly differ between groups (normosmia: 2.5 ± 2.9; hyposmia: 4.3 ± 4.2; functional anosmia: 3.0 ± 5.2; *p* = 0.17).

Comparison of normosmic PD patients and controls showed better olfactory thresholds at all turning points in patients than controls. A further two-way RM-ANOVA on normosmic subjects (PD: *N* = 8; controls: *N* = 60) showed significant effect of Group (*F*_[1,66]_ = 14.9; *p* < 0.001), Turning Point (*F*_[6,396]_ = 19.1; *p* < 0.001) and significant Turning Point × Group interaction (*F*_[6,396]_ = 4.1; *p* = 0.001). Post hoc analyses showed that the detection threshold was significantly higher (i.e., better function) in normosmic PD patients in comparison to controls at all turning points except the last one (*p* ranging from < 0.001 to 0.003; Fig. 3B). The difference between the first turning point and the final detection threshold score (i.e., the mean of the last four turning points) significantly differed between normosmic PD patients (2.5 ± 2.9) and controls (0.3 ± 2.6; *p* = 0.026).

### Threshold test trajectories analysis in PD according to motor and pharmacological variables

Two-way RM-ANOVA showed no effect of H–Y (*F*_[1,56]_ = 0.3; *p* = 0.61), UPDRS-III (*F*_[1,56]_ = 0.3; *p* = 0.62), or LEDD (F_[1,56]_ = 0.1; *p* = 0.79) and no Turning Point × H–Y (*F*_[6,336]_ = 2.7; *p* = 0.69), Turning Point × UPDRS-III (*F*_[6,336]_ = 0.3; *p* = 0.93), or Turning Point × LEDD (*F*_[6,336]_ = 0.7; *p* = 0.95) interaction.

### Threshold test trajectory analysis in PD according to cognitive status

Olfactory thresholds were better in PD patients without vs. those with visuospatial function deficits. Two-way RM-ANOVA showed significant effect of Visuospatial Function (*F*_[1,48]_ = 4.8; *p* = 0.031) and significant Turning Point × Visuospatial Function interaction (*F*_[6,342]_ = 2.8; *p* = 0.017), while the other factors were not significant. Post hoc showed that detection threshold value was significantly lower in PD patients with vs. without visuospatial function deficits at the first (*p* = 0.002) and second turning point (*p* = 0.004; Supplementary Fig. 2). The difference between the first turning point and the final threshold score significantly differed between PD patients without (4.2 ± 4.5) vs. those with visuospatial dysfunction (1.3 ± 2.5; *p* = 0.029).

Two-way RM-ANOVA including MCI and the other cognitive domains (except language, because no patient had language domain dysfunction) as between-group factors yielded neither significant effect of cognitive status nor significant interaction with Turning Point. The difference between the first turning point and the final threshold score did not significantly differ in PD patients according to the presence/absence of MCI and involvement of the other cognitive domains.

### Threshold test trajectories analysis in PD according to neuropsychiatric variables

Two-way RM-ANOVA showed no effect of HAD (*F*_[1,56]_ = 1.7; *p* = 0.20) or AES-S (*F*_[1,56]_ = 0.4; *p* = 0.52) and no Turning Point × HAD (*F*_[6,336]_ = 0.1; *p* = 0.77) or Turning Point × AES-S (*F*_[6,336]_ = 0.3; *p* = 0.59) interaction.

## Discussion

This study, for the first time, explored a threshold test trajectories analysis and suggested an underlying possible adaptation phenomenon in PD patients in comparison to healthy controls. The new findings of the study were: (a) MA-PD patients showed a different pattern of turning points trajectory (i.e., faster threshold adaptation) in comparison to controls and this result was confirmed in the whole PD group (i.e., MA-PD and OA-PD), having age no effect on our findings; (b) overall olfactory function influenced the threshold trajectory pattern (i.e., worse threshold at all turning points in subjects with worse overall olfaction) in PD patients; (c) normosmic PD patients showed a different pattern (i.e., better thresholds at all turning points) in comparison to normosmic controls; (d) cognitive function had limited effect on our findings, being visuospatial dysfunction the only factor significantly influencing the olfactory threshold measurements; and (e) motor, pharmacological and neuropsychiatric variables did not influence our findings.

At variance with some previous studies (Hedner et al. 2010; Rahayel et al. 2012; Whitcroft et al. 2016; Cecchini et al. 2019), but in keeping with other reports (Quinn et al. 1987; Bovi et al. 2010; Park et al. 2018; Masala et al. 2018), we found detection threshold final score to be worse in PD patients than controls. Different PD clinical features across studies might explain these discrepancies. Alternatively, differences in olfactory threshold trajectories might have contributed to the previous heterogeneous findings on detection threshold final score, which is calculated as the mean of the last four out of seven turning points, in PD patients. These data support the view that exploring the whole olfactory threshold trajectory could offer complementary information on odor threshold dysfunction in patients with chemosensory impairment due to different causes (Chen et al. 2020).

We observed abnormal olfactory threshold trajectories, suggesting faster olfactory adaptation, in PD compared to controls. In detail, PD patients showed better olfactory detection threshold performance than controls at the first two turning points of the threshold trajectories, then they rapidly declined and scored worse than controls at the last turning points, showing a reverse pattern over time. Moreover, the difference between the first turning point value and the final threshold score in PD patients was significantly larger than controls, supporting a possible faster adaptation phenomenon in PD patients.

Our data agree with those on patients with olfactory deficit due to different causes and extend the finding to PD (Chen et al. 2020). Besides, in keeping with Chen et al. (2020), we found overall olfactory *status* to have strong influence on olfactory threshold trajectories in PD. Indeed, functional anosmic patients showed worse performance than hyposmic and normosmic ones, but the number of trials did not significantly differ when comparing PD patients to controls, probably due to the low number of subjects.

Olfactory adaptation might be related to different physical and chemical properties of the odorant itself (Dalton 2000). Hence, the trajectories pattern here observed might be specific to n-butanol and other odorants might yield different results in patients and controls. In addition, some trace elements of ambient air may be inhaled during SSET (Williams and Ringsdorf 2020) and n-butanol is an abundant volatile organic compound in indoor air environment (Pacharra et al. 2020). Thus, a possible influence of these air elements on the olfactory receptors neurons (ORNs) cannot be excluded.

Besides, a recent in vivo invertebrate study suggested a new two-receptor olfactory model where both ORNs and the glial supporting cells cooperate promoting olfactory adaptation, highlighting the importance of the cross talk between these cells at the peripheral level (Duan et al. 2020). Indeed, olfactory adaptation has been suggested to reflect both the peripheral and the central nervous system structures (e.g., piriform cortex) involved in chemosensory processing (Iannilli et al. 2017; Pellegrino et al. 2017). Both neurodegeneration and aging may affect olfactory neuroepithelium that might become irregular and patchy and could be replaced by respiratory epithelium (Child et al. 2018). In this regard, a very recent human in vivo study showed alpha-synuclein pathological aggregates in olfactory mucosa samples since the prodromal PD stages in association with olfactory deficit (Stefani et al. 2021), highlighting the early peripheral involvement of the olfactory system in PD. Therefore, the reverse olfactory threshold trajectory pattern in PD might be due to the possible reduced number of surviving ORNs that are rapidly occupied by odorant molecules and become dysfunctional and/or to a functional deficit of the olfactory mucosa supporting cells, and this phenomenon could be influenced by the olfactory *status* that is directly related to the number of ORNs (Tian et al. 2016; Chen et al. 2020).

MA-PD patients showed abnormal olfactory threshold trajectories compared to age- and sex-matched controls. This different pattern of olfactory thresholds was confirmed in OA-PD patients and age was not a significant covariate in multivariate models. These findings suggest that, despite the age-related olfactory neuroepithelial changes (Child et al. 2018), olfactory threshold adaptation might be a promising psychophysical measure apparently not influenced by age in PD patients. These findings warrant replication in future larger studies.

Furthermore, we may speculate that fatigue, a frequent non-motor feature of PD (Kluger et al. 2016; Masala et al. 2018), might have also contributed to our findings, since the threshold test is driven with the patient blindfolded and lasts 15–20 min, a possibly fatiguing condition (Oleskiewicz et al. 2017). Future studies with suprathreshold stimuli (Tavassoli and Baron-Cohen 2012) should include data on fatigue to better test this hypothesis. We rule out the hypothesis that attention might have influenced our findings because performance in this cognitive domain did not influence our findings.

In the normosmic PD group, we found higher detection threshold values (i.e., better olfactory detection performance) than controls at all turning points, except the last one, suggesting they may act as “supersensors”, especially in the first trials, likely because of still unexplored peripheral and/or central compensatory mechanisms (Pellegrino et al. 2017). To the best of our knowledge, this is a new finding adding to the currently limited knowledge on normal olfactory function in PD. Data on normosmic PD patients are controversial and still debated. Whereas one study suggested normosmic PD to represent a unique clinical phenotype with a more benign course (Lee et al. 2015), another study found no differences between normosmic and hyposmic PD patients (Rossi et al. 2016). Indeed, normosmia in PD is rare (Haehner et al. 2009), and the small number of cases reported to date represents a limitation prompting future multi-center studies to better explore olfactory function in this subgroup of PD patients.

Cognitive function had limited effect on olfactory trajectories pattern in PD patients, in keeping with the view that odor threshold is a low-level perceptual process and carries lower cognitive load than odor identification and discrimination (Dulay et al. 2008; Hedner et al. 2010; Rahayel et al. 2012; Whitcroft et al. 2016). Only visuospatial dysfunction significantly influenced the first turning point and the detection threshold score, in keeping with our previous study, where we found worse olfactory function in PD patients with visuospatial dysfunction (Cecchini et al. 2019). However, the limited number of patients with the involvement of this cognitive domain suggest caution with the interpretation of this finding, which should be confirmed in larger studies. Interestingly, visuospatial and olfactory dysfunction were reported to share some pathological grounds, namely parietotemporal and limbic areas metabolic and electroencephalographic changes (Iannilli et al. 2017). From a clinical perspective, our data suggest that olfactory threshold trajectories analysis may represent a new time-saving psychophysical approach that may be applied also to PD patients with some degree of cognitive dysfunction, being this test less cognitively demanding than odor identification or discrimination (Hedner et al. 2010; Rahayel et al. 2012; Cecchini et al. 2016).

In accordance with previous reports (Doty et al. 1992; Rossi et al. 2015; Fullard et al. 2017), PD motor, pharmacological and neuropsychiatric variables did not influence olfactory threshold trajectories, suggesting the feasibility of this analysis in patients with different PD clinical features and treatments.

The present study has some limitations. First, the SSET threshold test may not represent the optimal psychophysical test to investigate olfactory adaptation (Chen et al. 2020), and future studies should confirm these findings with suprathreshold stimuli presented for longer amounts of time (Tavassoli and Baron-Cohen 2012) and with the assessment of the recovery curve (Pellegrino et al. 2017). Indeed, from a clinical perspective, psychophysical paradigms to assess suprathreshold olfactory adaptation could be useful, but they are time-consuming, while the present olfactory threshold trajectories analysis can be derived from SSET and other validated threshold tests based on the staircase technique and is feasible in elderly and mildly cognitively impaired patients, who may experience difficulties with more complex tests. Second, we performed a threshold analysis based on a single odorant, i.e., n-butanol, which activates both olfactory and trigeminal systems (Foguem et al. 2018), like most odorants do (Doty et al. 1978), and the interaction between these systems (Tremblay and Frasnelli 2018) may account for some pathophysiological specificities of PD-related olfactory loss than other olfactory dysfunction types. Indeed, a specific pattern of trigeminal responsiveness was recently reported in PD (Tremblay et al. 2019). Other factors, such as relevance, pleasantness and psychophysical features of odorant, and gender, have been reported to influence adaptation (Stone et al. 1972; Jacob et al. 2003; Kobayashi et al. 2008), and the present findings should be confirmed with other odorants. Third, our conclusions are not supported by neuropathological, neurophysiological or neuroimaging data that could further reinforce our reasoning on the anatomical bases of the present psychophysical data.

In summary, we found different olfactory threshold trajectory patterns in PD than controls, suggesting a possible faster adaptation phenomenon in PD patients, with no influence of age and cognitive function, and we offered some new very preliminary insights on normosmic PD patients, which seem to represent a specific subgroup. Olfactory threshold trajectories analysis is a feasible psychophysical approach that may offer interesting and complementary information to SSET and should be explored and validated in larger prospective studies.

## Supplementary Information

Below is the link to the electronic supplementary material.

Supplementary Fig. 1. Flow diagram of the study and reasons for patients’ exclusion (JPG 413 KB)

Supplementary Fig. 2. Olfactory threshold at the seven turning points (TP) in patients with Parkinson’s disease (PD) without (VS-; N = 50; open boxes) and with visuospatial dysfunction (VS + ; N = 9; closed boxes). Higher and lower olfactory threshold value represents better and worse performance, respectively. *Marks significant VS- vs. VS + comparison (JPEG 172 KB)

## Data Availability

Anonymised data used for this study are available from the corresponding authors on reasonable request.
