# Supplementary figures and images for: Olfaction in patients with Parkinson’s disease: a new threshold test analysis through turning points trajectories
Source: J Neural Transm (Vienna). 2021 Jul 30;128(11):1641–53. doi: 10.1007/s00702-021-02387-z (PMC8536637; doi:10.1007/s00702-021-02387-z)

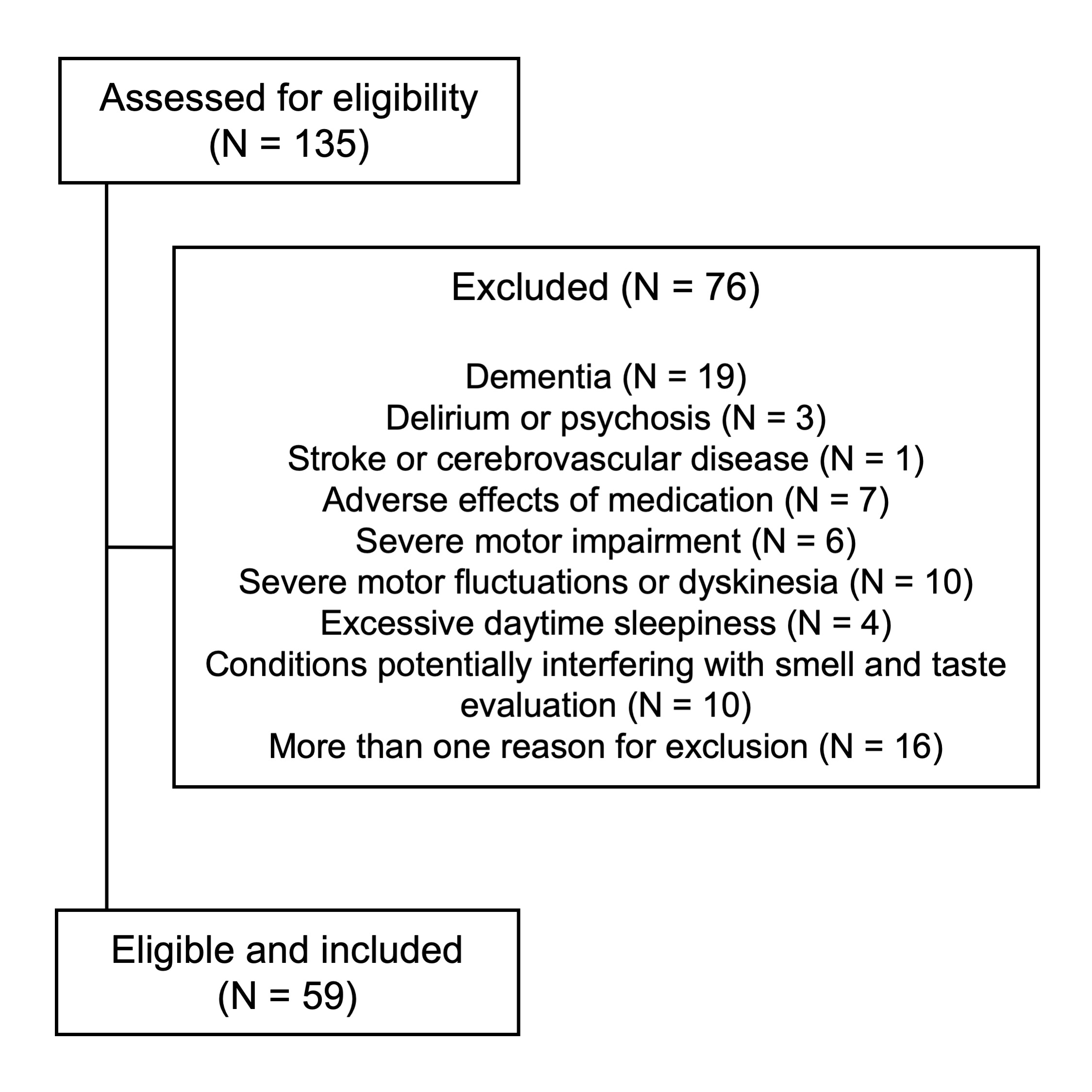

Supplement: Supplementary file 1 — Supplementary Fig. 1. Flow diagram of the study and reasons for patients’ exclusion (JPG 413 KB) [file 702_2021_2387_MOESM1_ESM.jpg]

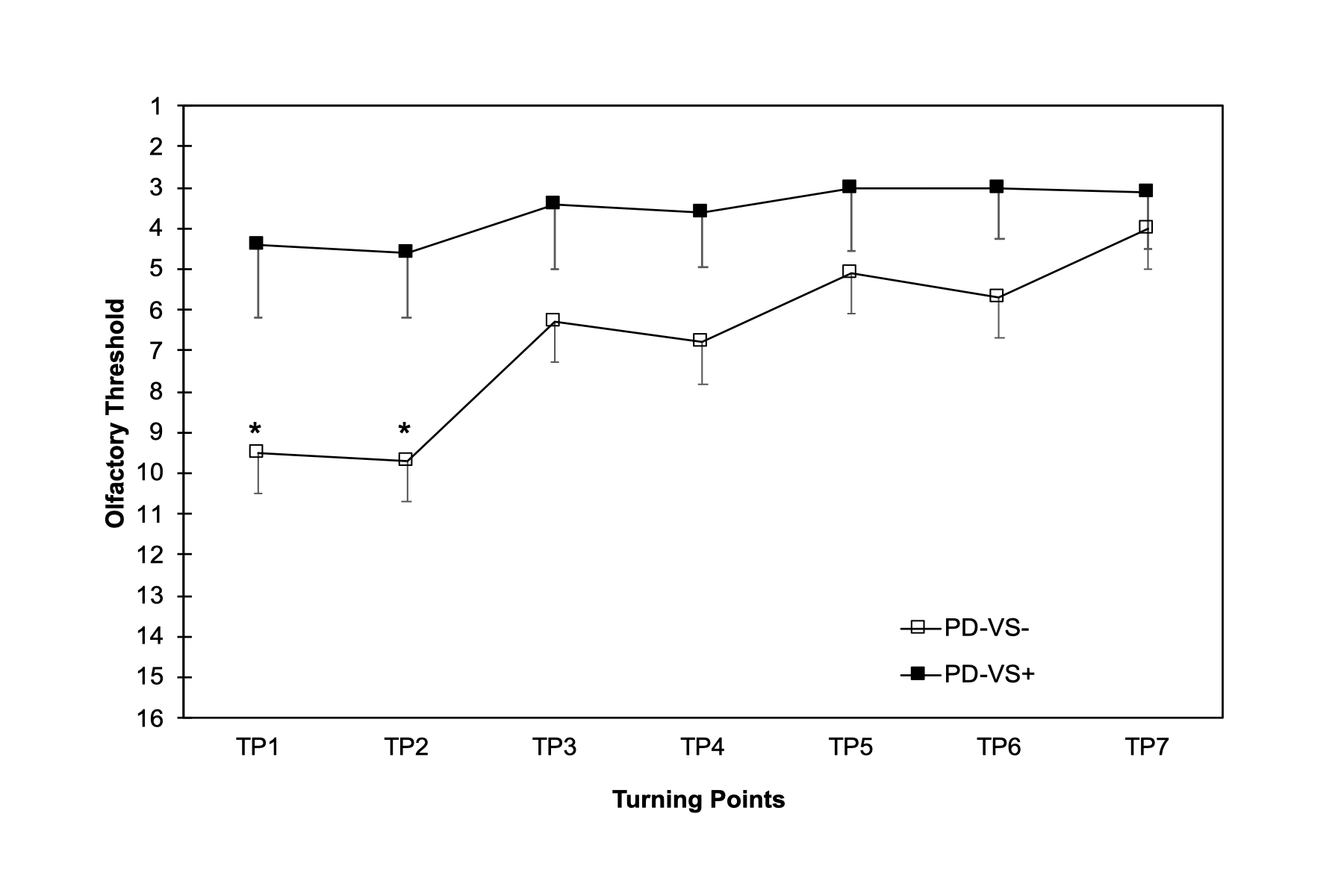

Supplement: Supplementary file 2 — Supplementary Fig. 2. Olfactory threshold at the seven turning points (TP) in patients with Parkinson’s disease (PD) without (VS-; N = 50; open boxes) and with visuospatial dysfunction (VS + ; N = 9; closed boxes). Higher and lower olfactory threshold value represents better and worse performance, respectively. *Marks significant VS- vs. VS + comparison (JPEG 172 KB) [file 702_2021_2387_MOESM2_ESM.jpeg]
